# How can diagnostic assessment programs be implemented to enhance inter-professional collaborative care for cancer?

**DOI:** 10.1186/1748-5908-9-4

**Published:** 2014-01-03

**Authors:** Anna R Gagliardi, Terri Stuart-McEwan, Julie Gilbert, Frances C Wright, Jeffrey Hoch, Melissa C Brouwers, Mark J Dobrow, Thomas K Waddell, David R McCready

**Affiliations:** 1 Toronto General Research Institute, University Health Network, Toronto, Canada; 2 Gattuso Rapid Diagnostic Centre and Solid Tumour Oncology, University Health Network, Toronto, Canada; 3 Planning and Regional Programs, Cancer Care Ontario, Toronto, Canada; 4 Odette Regional Cancer Centre, Sunnybrook Health Sciences Centre, Toronto, Canada; 5 Pharmacoeconomics Research Unit, Cancer Care Ontario, Toronto, Canada; 6 Department of Oncology, McMaster University, Hamilton, Canada; 7 Cancer Services and Policy Research Unit, Cancer Care Ontario, Toronto, Canada; 8 Surgery, University Health Network, Toronto, Canada; 9 Surgical Oncology, University Health Network, Toronto, Canada

**Keywords:** Inter-professional collaborative care, Multidisciplinary care team, Inter-professional relations, Communication, Cooperative behavior, Diagnostic assessment program, Breast cancer, Lung cancer

## Abstract

**Background:**

Inter-professional collaborative care (ICC) for cancer leads to multiple system, organizational, professional, and patient benefits, but is limited by numerous challenges. Empirical research on interventions that promote or enable ICC is sparse so guidance on how to achieve ICC is lacking. Research shows that ICC for diagnosis could be improved. Diagnostic assessment programs (DAPs) appear to be a promising model for enabling ICC. The purpose of this study was to explore how DAP structure and function enable ICC, and whether that may be associated with organizational and clinical outcomes.

**Methods:**

A case study approach will be used to explore ICC among eight DAPs that vary by type of cancer (lung, breast), academic status, and geographic region. To describe DAP function and outcomes, and gather information that will enable costing, recommendations expressed in DAP standards and clinical guidelines will be assessed through retrospective observational study. Data will be acquired from databases maintained by participating DAPs and the provincial cancer agency, and confirmed by and supplemented with review of medical records. We will conduct a pilot study to explore the feasibility of estimating the incremental cost-effectiveness ratio using person-level data from medical records and other sources. Interviews will be conducted with health professionals, staff, and referring physicians from each DAP to learn about barriers and facilitators of ICC. Qualitative methods based on a grounded approach will be used to guide sampling, data collection and analysis.

**Discussion:**

Findings may reveal opportunities for unique structures, interventions or tools that enable ICC that could be developed, implemented, and evaluated through future research. This information will serve as a formative needs assessment to identify the nature of ongoing or required improvements, which can be directly used by our decision maker collaborators, and as a framework by policy makers, cancer system managers, and DAP managers elsewhere to strategically plan for and implement diagnostic cancer services.

## Background

### Need for collaborative cancer care

Most cancer patients require multimodal assessment and treatment including radiologic and pathologic detection, confirmation and characterization, and surgery, chemotherapy, and/or radiation for cure or palliation [1]. Following initial treatment, patient needs vary as they undergo follow-up surveillance to detect recurrent or secondary cancer, with many facing physiological and psychosocial difficulties as a result of their cancer and/or its treatment [2]. In addition, most cancer patients require management to prevent or treat co-morbid conditions [3]. Thus, cancer management is complex, and compounded by the fact that multimodal care is delivered by different professionals, in different settings, and at different time points. Research has established that coordinated, collaborative service delivery improves clinical (*i.e*., mortality, length of stay, readmission) and patient-reported (*i.e*., satisfaction, health related quality of life) outcomes for a variety of acute and chronic conditions including cancer [1,4,5]. This concept of inter-professional collaborative care (ICC) requires ongoing interaction among various types of health professionals to assess, plan, negotiate, provide, and review care for individual patients [6].

### Barriers of collaborative cancer care

It has been proposed that one-third of cancer cases could be prevented, another third cured, and the rest effectively treated if management consistently complied with existing guidelines [7]. Most cancer management guidelines recommend ICC but do not specify how this can be achieved [8]. A non-systematic review of the literature on ICC in cancer found that formal policies and structures improved treatment decisions, implementation of treatment decisions, documentation of treatment decisions, attendance at joint meetings, professional diversity at meetings, completeness of information presented at meetings, management according to guideline recommendations, time to diagnosis or treatment, survival, role identification among team members, team effectiveness, and staff wellbeing [9]. However, timely and appropriate ICC was challenged by many patient, provider, team, and system level factors [10]. Other barriers included strategic differences across organizations, limited administrative support and identified leads for the collaborative process, and organizational and individual provider reluctance to share resources and power [11]. Given multiple associated benefits, efforts are needed to promote and support ICC for the clinical management of cancer patients. First, improved understanding of which ICC approaches lead to improved patient, provider and organizational or system outcomes is required so that we can meaningfully evaluate whether and how cancer patients experience ICC [12,13]. Further understanding of how various ICC models lead to beneficial patient, provider, institutional, and health system outcomes will provide insight on when and in what way to implement these models.

### Our research on collaborative cancer care

We have jointly conducted several research studies that identified numerous challenges of ICC for cancer, and evaluated the availability and impact of interventions to support ICC. ARG surveyed and interviewed Ontario clinicians and managers involved in cancer care across several studies. Participants identified numerous ICC challenges, such as timely access to testing for diagnosis and staging, lack of human and technical resources, identifying and communicating with specialists, coordinating referral to and back from specialists, confusion among multidisciplinary team members about who was to coordinate management, and the need for system level support [14-19]. Interventions to support ICC suggested by participants included patient held medical records, cancer specific medical record, standardized referral and reply forms, centralized cancer diagnostic facilities, regional outreach clinics, and use of telemedicine. ARG conceptually analyzed the literature to describe models of ICC [20]. Determinants of positive objective and subjective patient, team and organizational outcomes included system or organizational support, team structure, and team processes. ARG reviewed empirical research evaluating ICC for cancer patients [20]. Twenty-two studies of mixed design published between 2001 and 2009 were eligible. The majority of studies (17/22) assessed the role of general practitioners and supportive/palliative care workers in cancer patient follow-up. Five of 22 studies evaluated ICC for diagnosis or treatment decision making. Apart from tumor boards, no studies described interventions to enable ICC. Collectively this research suggests that most cancer providers function through parallel or consultative, rather than integrated models of care.

FCW spearheaded several investigations to describe and evaluate multidisciplinary cancer conferences (MCCs) as an intervention to support ICC. Also known as tumor boards, these are defined as regularly scheduled meetings where healthcare providers discuss the treatment of individual cancer patients [1]. First, she chaired a multidisciplinary panel to issue an evidence- and consensus-based guideline describing MCCs [1]. FCW conducted a systematic review of the literature to examine the impact of ICC on clinical outcomes [21]. Twelve studies of various design reported statistically significant association between ICC and survival. General surgeons were surveyed to identify individual and organizational barriers to MCC adoption [22]. Surgeons said that MCCs were not well supported institutionally or widely accessible, few had a designated coordinator, and most reviewed only rare or select cases rather than all new cancer patients. Interviews and observation were used to explore MCC use in four hospitals [23]. Thirty-seven MCCs were observed at three hospitals, and 48 clinicians and administrators were interviewed. Institutions lacked the capacity to fully implement MCCs as part of routine practice.

MJD developed a measure of cancer services integration and conducted a population-based survey of Ontario health professionals to evaluate integration. The study identified 12 factors that accounted for the majority of variation in cancer services integration [24]. This work emphasized how leadership, coordination, resource allocation, and communication influence overall integration of cancer services. Further analysis of this data revealed variability in access to electronic health records (EHRs) across different provider groups, organization types, and geographic locations, which may limit ICC [25]. Another analysis focused specifically on the benefits of MCCs as a model of ICC [26]. Overall, 74% of respondents were aware of MCCs within their region, but only 58% were regular participants.

### Diagnostic assessment programs

MCCs represent one model of ICC for treatment decision making. The time interval from suspicion to diagnosis of cancer involves numerous consultations and testing, and is a confusing time for patients. Timely diagnosis can lead to improved access to MCCs or other consultation, earlier treatment and a better prognosis [1-21]. Clinicians and managers suggested the need to improve ICC earlier in the cancer trajectory given barriers of access to, and coordination of diagnosis and staging, and recommended centralized diagnostic facilities [14-19]. An expert panel assembled by the provincial cancer agency issued organizational standards for Diagnostic Assessment Programs (DAPs) to coordinate diagnostic tests and integrate multidisciplinary expertise [27,28]. A summary of recommended DAP features appears in Table 1.

**Table 1:** DAP standards

**Component**	**Description**
Team composition	Administrative
	• Director/manager
	• Reception, clerical and bookings
	Health professionals
	• Assessment coordinators (examples):
	• Radiologists
	• Pathologists
	• Primary care
	• Psychosocial support
	Specialists
	• Surgeon specialists
	• Respirologists (lung)
	• Endoscopists (colorectal and other)
	Technicians
	• Ultrasound technologists
	• Mammographers (breast)
Scope of diagnostic activity	Examination
(diagnostic activity differs depending on disease site)	• Physical exam
	• Other disease site specific
	Imaging, diagnostic and staging procedures
	• Ultrasound
	• MRI
	• X-ray
	• CT scan
	• PET
	• Upper endoscopy
	• Colonoscopy
	• Bronchoscopy
	• Cystoscopy
	• Bone scan
	• Mammography
	• Other disease site specific
	Surgical consultation and procedures
	• Biopsy
	• Fine needle aspiration cytology
	• Biopsy
	Pathology and laboratory medicine
	• Standardized surgical pathology requisition forms
	• Routine analysis and pathology reporting
	• Special pathological studies such as markers, flow, molecular, etc
	• Clinical lab testing of tumour markers, hematology, etc.
	Supportive care
	• Education/psychosocial support
	• Dietetics
	• Genetic counselling
	• Other supportive services
• Access	• Regionalized, centralized
	• One stop
	• Virtual
• Entry point	• Primary care providers or specialist
	• Screening program
	• Self referral
• Operational features	• Entry
	• Fast access booking
	• Priority booking
	• Open-access booking
• DAP core elements	• Assessment coordinator
	• Multidisciplinary care conference (MCC) team/treatment team
	• Cross-DAP collaboration
• Provincial indicators of quality for cancer DAPs	• Time intervals
	• Clinical outcomes
	• Quality of care
	• Patient satisfaction
• Guidelines, standards and services frameworks	• Guidelines and service frameworks for primary care providers
	• Evidence-based investigative algorithms and guidance documents
	• Wait-times benchmarks

### Study rationale

ICC for cancer leads to multiple system, organizational, professional, and patient benefits. However, our analysis of conceptual literature did not reveal optimal ways to achieve ICC, and our review of empirical literature revealed that no interventions apart from MCCs have been used to promote ICC for cancer care. Our research with health professionals identified limited support for, use of, and access to interventions that enable ICC for cancer, particularly outside of designated cancer hospitals in community settings where the majority of cancer care takes place, and for cancer diagnosis. DAPs appear to be a promising model by which to enable ICC. In 2007, the provincial cancer agency funded four pilot DAPs and all achieved reductions in wait times (http://www.cancercare.on.ca/pcs/diagnosis/diagprograms/). Hospital one reduced median time from suspicion of breast cancer to biopsy by 60% (38 to 15 days) and from suspicion to diagnosis by 53% (42 to 20 days); hospital two reduced time from referral to colonoscopy for patients with positive fecal occult blood test results by 78%; hospital three reduced lung cancer wait times from 113 to 69 days for referral to diagnosis; and hospital four reduced lung cancer wait times from 120 to 44 days for suspicion to diagnosis.

Wait times are only one possible outcome of ICC. We require more information about how to design and implement DAPs to optimize ICC and achieve the range of associated beneficial outcomes. We conducted a systematic review of the cancer literature to describe clinical and economic evaluations of DAPs [29]. Most of the 20 eligible studies did not base their evaluations on guideline recommendations or quality indicators, or include economic evaluations. Several DAPs were implemented across Ontario, so more comprehensive evaluation was warranted and possible to better understand how various DAP models enabled ICC. The purpose of this study is to:

1. Describe DAP structure, function and outcomes according to published DAP standards, clinical guideline recommendations, and a theoretical framework of ICC.

2. Conduct a pilot costing analysis of delivering diagnostic services with DAPs.

3. Explore challenges of DAP implementation and operation, and associated ICC.

4. Issue recommendations that may optimize the implementation, operation and outcomes of DAPs.

This research will not evaluate DAPs from the perspective of patients. While crucial, that objective warrants separate multi-year investigation to explore patient preferences for diagnostic care first through review of the literature, then by interviewing patients with various characteristics who did and did not experience DAP care. This would establish patient-informed performance measures of diagnostic services and ICC, which do not exist. We found that patient views about cancer care performance measures differed from those of health professionals, thus development of patient-informed performance measures is necessary to fully evaluate the services provided by DAPs and the degree to which the DAP model enables ICC [30]. Instead, this proposal responds to the expressed needs of our research partners, and for multiple reasons including feasibility, focuses first on evaluating DAPs according to evidence-based standards for DAPs and clinical care delivery, and by soliciting the views of involved health professionals. This preliminary evaluation is needed to establish a baseline understanding of how DAPs were implemented and function. Only then will we have sufficient insight on factors influencing DAP outcomes that we could ask patients about, and an established relationship with DAP collaborators to enable an expanded research study that would include patient recruitment.

## Methods

### Design

A case study approach was chosen to explore multiple factors that influence ICC including DAP structure, processes and outcomes [31]. These will be assessed according to DAP standards and guideline recommendations for staging and diagnosis of breast [32-34] and lung cancer [27,28]. This will enable comparison between ‘cases’ (DAPs) that vary by type of cancer, academic status, and geographic region. This may identify whether and how differences in DAP leadership, staffing, resources, and referral patterns influence ICC, or whether ICC must be enabled differently by condition. This approach is suitable for examining complex issues that require holistic interpretation (‘triangulation’) of data collected in a variety of ways from different sources. Data will be collected from medical records and interviews. Four hospitals from different regions of Ontario agreed to participate. Two are considered academic teaching hospitals. Each site features a breast DAP and a lung DAP, for a total of eight participating DAPs. This study was reviewed and approved by the University Health Network Research Ethics Board, and research ethics boards at each of the four participating hospitals.

### Conceptual framework

**Figure 1:**
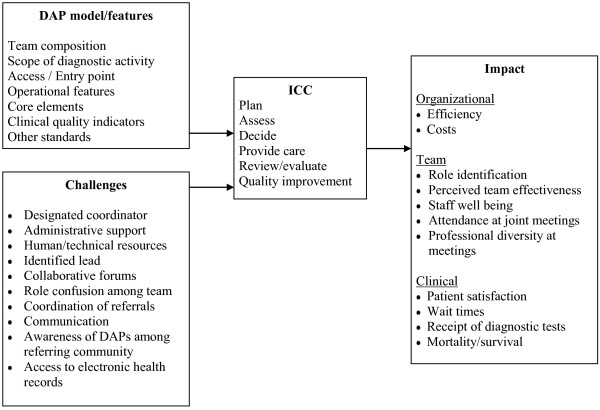
Conceptual framework describing factors that influence how DAPs achieve ICC and associated benefits.

The overall goal of this study is to explore how DAPs enable ICC and associated outcomes. ICC was defined as ‘interaction among various types of health professionals to plan or evaluate services, or plan, provide or review results or outcomes for individual patients’. There is no single existing model or theory that describes factors influencing the quality of ICC, so we compiled a conceptual framework from several sources. We had reviewed several bodies of knowledge describing models of health professional interaction for patient management according to the concepts of teamwork, inter-professional collaboration, continuity of care, integrated service delivery, inter-organizational collaboration and case management, and extracted data on common domains and elements, and associated outcomes [20]. This generated core components and enablers of ICC that were common across the models. Organizational standards for DAPs that are described in Table 1[27,28] were mapped onto this preliminary framework. The conceptual framework was expanded by adding challenges [10-26] and beneficial outcomes [9-21] identified in our background review of the literature. We also reviewed clinical guidelines for breast [32-34] and lung cancer [27,28] to incorporate elements of desirable care delivery and outcomes. The resulting conceptual framework (Figure 1) will be used to inform the development of data collection instruments and data analysis. Findings will be used to validate and extend the conceptual framework to describe how DAP structure, function and support enable ICC, and associated outcomes.

### Medical record review to describe DAP services and outcomes

#### Approach

To describe DAP function and outcomes, and gather information that will enable costing, recommendations expressed in DAP standards and clinical guidelines will be assessed through retrospective observational study. Data will be acquired from databases maintained by participating DAPs and the provincial cancer agency, and confirmed by and supplemented with review of medical records.

#### Sampling

Eligible patients include those 18 years of age or older with suspected lung or breast cancer who were referred to participating DAPs between 1 January 2012 and 31 December 2012. Based on input from collaborating sites, we initially estimated that this includes a mean of 15 new patients per month by two types of cancer in four sites for an annual total of 360 patients per site, and an overall total of 1,440 patients. Assuming a type I error (alpha) rate of 0.05, power of 0.80, and equal sample sizes for two comparative groups (*i.e*., academic/community status or breast/lung cancer), 170 patients (85 in each group) in total would be required to detect a statistically significant difference in compliance with a given DAP or clinical guideline standard of 15%. Thus, estimated patient sampling is more than sufficient to identify variations in process or outcome performance measures according to varying DAP features. However, DAPs vary in case volume. To give equal weight to each DAP at each participating site based on patient volume, 15% of patients will be randomly sampled from among those newly referred during the given time period. For all sites, this is equal to or greater than minimal sample sizes estimated by traditional power calculation.

### Data collection and analysis

Data reflecting DAP and guideline recommendations for diagnostic activity (Table 1) will be extracted from medical records. A trained data abstractor will visit participating sites. Before this, a data extraction form will be developed and independently pilot tested on five cases by two individuals. They will compare congruence of extracted data to assess how the form should be revised. This will be repeated until the format and content of the data extraction form is satisfactory and congruence of independently extracted data is high. During chart review, 5% of charts will be re-abstracted for data quality monitoring. Summary statistics will be used to report compliance with DAP and guideline standards for patients overall and by DAP, academic status, and type of cancer. Statistical significance of differing outcomes will be reported with the Pearson’s chi-square test. A generalized linear mixed model approach will be used to address binary and continuous process and outcome variables. Hierarchical modeling (patient level one, hospital level two) will be used to allow for clustering by hospital.

### Pilot costing analysis of DAP diagnostic visits and services

#### Approach

Our ultimate goal is to conduct a cost-effectiveness analysis (CEA), but this is a complex undertaking requiring considerable data on the actual number and nature of services provided per patient and their cost. The purpose of CEA is not hypothesis testing, but rather it is estimation [35]. To do a CEA, one needs to compute estimates of the extra cost (∆C) and the extra effect (∆E) of an intervention. The ratio of ∆C to ∆E is called the incremental cost-effectiveness ratio (ICER) and is the main statistic in CEA. Such data are not readily available in administrative databases and often requires primary data collection from medical records. We currently do not know whether data required for a CEA of DAPs is available in medical records. Therefore, we will conduct a pilot study to explore the feasibility of estimating the ICER using person-level data from medical records and other sources. This will allow us to prepare for a future more comprehensive CEA of DAPs across multiple sites.

#### Sampling

Because this is a pilot study, we will focus our efforts on one breast and one lung DAP at one site to examine whether and how data routinely collected in medical records can be used as the main source of data for costing. This site was chosen because they have collected DAP-specific data for several years and used it for internal reporting, and the site is easily accessible to investigators, which facilitates data collection and minimizes study costs. The patient sample will be similar to that used for the retrospective observational study at this site.

### Data collection and analysis

As the purpose of this pilot costing analysis is to explore the potential for estimating costs using readily available program, hospital, and patient data, we will focus on data related to service use. Costs are calculated as ‘price’ times ‘quantity’, and the data we are collecting from patient charts are the ‘quantities’. The number and nature of all diagnostic services provided to each eligible patient will be extracted. This will be reviewed with collaborators to create a standardized list of defined, unique services, for which costs will be acquired from hospital sources and OHIP billing codes. The question of interest is whether the patient chart provides relevant data on health service use. By ‘costing’ the data in the charts, this produces a fuller estimate of Total Cost. We will use a paired t-test for each patient to test whether the Total Cost using the chart data is statistically significantly different from the Total Cost using data acquired from hospital or program sources.

### Key informant interviews

#### Approach

Telephone interviews will be conducted with health professionals, staff, and referring physicians from each DAP to learn about barriers and facilitators of ICC. Qualitative methods based on a grounded approach will be used to guide sampling, data collection, and analysis [36]. The theoretical framework will inform interview questions and their analysis.

#### Sampling and recruitment

Interviewees will be identified by the collaborating key contact for each DAP (known sponsor approach). One manager, nurse, and physician from each DAP, plus two referring physicians will be recruited, for a minimum target of 40 interviews, to collect information from individuals who vary by health profession and site sampling criteria (purposive sampling). They will be invited by regular mail and email, and asked to sign and return a signed consent form. Information from representative, rather than a large number of participants is needed in qualitative research. It is not meant to produce generalizable results, but to provide an in-depth exploration of issues. Sampling is concurrent with data collection and analysis, and proceeds until no further unique themes emerge (thematic saturation). If saturation is not achieved after 40 interviews, further interviews will be pursued with additional individuals identified by the key contact, and by interviewees (snowball sampling).

### Data collection and analysis

A semi-structured interview guide will be developed to explore how DAP structure, operation, and other factors influence or challenge ICC. Participants will be prompted to discuss system or organizational support, team structure, and team processes, and how these factors influence ICC and outcomes. The interview guide will be pilot-tested with one manager and one clinician from a DAP not participating in the study to refine the wording and flow of questions. Telephone interviews of approximately 30 minutes will be audio-recorded, and converted to text by a professional transcriptionist. Unique themes will be identified in an inductive manner using constant comparative technique [37,38]. Transcripts will be read independently by two individuals to identify, define, and organize themes. A log will be maintained of emerging codes, their definition, and sample narrative illustrating application of that code (open coding). The narrative will be reviewed (constant comparative technique) to identify all instances of the coding framework, and items not matching the framework, to determine how to expand or merge thematic codes (axial coding). The two will compare findings and achieve consensus through discussion. A third individual will resolve any conflicts. Coded text will be tabulated by theme and DAP to compare and interpret results.

## Discussion

As our population ages, an increase in the absolute number of cancer patients is expected. To improve their care and outcomes enhanced ICC is needed. The proposed study constitutes phase one of a longitudinal research program that will evaluate existing and alternative interventions to support ICC for cancer. It also represents the next phase in the evaluation of DAPs as a model by which to enable ICC following evaluation of diagnostic wait times for pilot program implemented across Ontario.

The proposed study may be limited in a number of ways. Medical record review will not be comprehensive of all cases at all DAPs across Ontario, and clinical measures are limited to wait times and receipt of basic diagnostic tests. However, the study is exploratory overall, and will attempt to identify whether particular DAP features that enable ICC appear to be associated with shorter wait times or more consistent receipt of diagnostic tests. If there are trends, this would warrant a future, larger-scale study to confirm such associations. Site sampling enhances the relevance of findings because we will include DAPs with a variety of features. Evaluation is informed by published and evidence-based DAP and clinical guidelines, and by a theoretical framework of ICC generated by review of several relevant bodies of knowledge. The costing component will establish a taxonomy of DAP visits and services, and associated costs that could be used by all DAPs to calculate costs, and in a future cost effectiveness study. The limitations of available data for cost effectiveness analysis are currently unknown, so we are taking a practical approach in conducting an exploratory/pilot costing analysis as the first step. Recruitment for qualitative interviews is always challenging, but we have identified a lead key informant at each site who will identify and link us with various health professionals and staff to achieve interviews. Interviews will be conducted with health professionals in various professional roles both internal and external to collaborating DAPs to enhance the depth and relevance of findings. Case study design triangulates data from multiple sources collected in multiple ways to generate in-depth information, which will be further integrated and interpreted through ongoing interaction with collaborators. A variety of additional factors enhance the feasibility and successful conduct of the proposed research. The research team, which has successfully collaborated on numerous previous studies, includes individuals with training, expertise, and experience in case studies, economic analyses, and qualitative methods (interviews, case studies), and experience in evaluating models of ICC. We were approached by project-specific partners to address their expressed information needs. This means they are interested in helping us to conduct the research, and will use the findings.

Multiple products and outcomes are expected. By interacting with various types of decision makers, we will identify barriers of ICC and associated suggestions for improvement. This may reveal opportunities for unique structures, interventions, or tools that enable ICC apart from MCCs or DAPs that could be developed, implemented, and evaluated through future collaborations between researchers and decision-makers. This study will describe DAP evolution and the extent of DAP implementation in Ontario according to compliance with standards, and feedback of stakeholders both internal and external to participating DAPs. This information will serve as a formative needs assessment to identify the nature of ongoing/required improvements, which can be directly used by our decision-maker collaborators, and as a framework by policy makers, cancer system managers, and DAP managers elsewhere to strategically plan for future services. Study findings will be shared with stakeholders representing difference professional roles and organizations from across Ontario to issue recommendations for DAP structure, implementation, and operation. Mechanisms by which to achieve ICC could then be better described in cancer guidelines and other tools that specify ICC. Cost-effectiveness analysis can establish a mechanism for evaluating the benefit of ICC as delivered by DAPs. Such modeling can be used by policy makers, cancer system managers, and DAP managers elsewhere. Our pilot costing exercise will be used to plan future cost-effectiveness studies. The study findings can be used to develop a theoretical framework of ICC, since our review of conceptual literature revealed the need for further development of measures by which to evaluate ICC. We and others can use this in future research. By identifying gaps in knowledge, we establish the need for additional primary investigation that describes current patterns of ICC and associated outcomes. By engaging with multiple stakeholders we develop relevant, feasible, and desirable interventions for enhancing DAP care, and more effectively exchange ideas and transfer the findings of this research to policy and practice.

## Competing interests

The authors declare that they have no competing interests.

## Authors’ contributions

ARG conceptualized and designed this study, and acquired funding. She will perform and/or oversee primary data collection, analysis, interpretation and report writing. TSM, TKW, DRM, and JH provided important guidance for the design and execution of the study. FCW, MCB, JH, MJD, JG, TSM, TKW, and DRM will participate in data analysis and interpretation. All co-investigators contributed to the preparation of this manuscript, and reviewed and approved the final version.
